# Biosorption of nickel and cadmium using *Pachira aquatica* Aubl. peel biochar

**DOI:** 10.1038/s41598-024-54442-w

**Published:** 2024-03-01

**Authors:** Talita L. S. Nascimento, Karine F. S. Oliveira, Joemil O. D. Junior, Alexandre S. Pimenta, Dulce M. A. Melo, Marcus A. F. Melo, Renata M. Braga

**Affiliations:** 1 Postgraduate Program in Chemical Engineering, Federal University of Rio Grande do Norte, Natal, 59078-970 Brazil; 2 Postgraduate Program in Materials Science and Engineering, Federal University of Rio Grande do Norte, Natal, 59078-970 Brazil; 3 Agricultural School of Jundiaí, Federal University of Rio Grande do Norte – UFRN, Macaíba, RN 59280-000 Brazil; 4 Postgraduate Program in Chemical, Federal University of Rio Grande do Norte, Natal, 59078-970 Brazil; 5 Chemical Engineering Department, Federal University of Rio Grande do Norte, Natal, 59078-970 Brazil; 6 Escola Agrícola de Jundiaí- UFRN, RN 160, Km 03, Distrito de Jundiaí, Macaíba, RN 59280-000 Brazil

**Keywords:** Biosorption, Heavy metals removal, Biomass, Biosorbent, Wastewater treatment, Environmental chemistry, Environmental impact

## Abstract

This study aimed to assess the value of Pachira aquatica Aubl. fruit peels by exploring their applicability in the biosorption process for the removal of Ni(II) and Cd(II) metal ions. The *Pachira aquatica* Aubl. fruit peel biochar (PAB) was extensively characterized through various techniques, including proximate analysis, helium pycnometer, XRD, SEM, point of zero charge determination, zeta potential measurement, and Boehm titration. Subsequently, kinetic, isotherm, and thermodynamic batch biosorption studies were conducted, followed by column biosorption tests. The characteristics of PAB, including low moisture content, a neutral point of zero charge, porosity, an irregular and heterogeneous structure, a negatively charged surface, and the presence of functional groups, indicate its remarkable capacity for efficiently binding with heavy metals. Biosorption equilibrium time was achieved at 300 min for both ions, fitting well with a pseudo second-order kinetic model and Langmuir isotherm model. These data suggest that the biosorption process occurred chemically in monolayer. The column C presented an exhaust volume of 1200 mL for Ni(II) and 1080 for Cd(II) and removal of 98% and 99% of removal for Ni(II) and Cd(II), respectively. In summary, PAB demonstrates substantial potential as a biosorbent for effectively removing heavy metals, making a valuable contribution to the valorization of this co-product and the mitigation of environmental pollution.

## Introduction

Chemical contamination of the environment by heavy metals is becoming increasingly serious worldwide due to human activities and industrial development^1^. Ni(II) and Cd(II), besides Pb(II), have been listed as priority pollutants by the US Environmental Protection Agency (EPA) and French Water Agency^2,3^. Due to their potential carcinogenicity, high toxicity, even at low concentration, long residence times, bioaccumulation and their non-decomposition by chemical or biological processes^4,5^

Therefore, environmental monitoring agencies set maximum permitted limits for heavy metals in the water to protect human health and the ecosystem. In Brazil, for instance, the CONAMA N°430/2011^6^ establishes that wastewaters containing heavy metals such as Ni(II) and Cd(II) must present a maximum of 2.0 mg/L and 0.2 mg/L, respectively, to be discharged into water bodies. Considering that, the concentration of Ni(II) and Cd(II) in an industrial effluent can reach 183 mg/L and 63 mg/L, respectively^7^, it is essential to use advanced techniques that can remove heavy metals from wastewater.

The most widely used technologies for this purpose are chemical precipitation, membrane filtration, coagulation and flocculation, ion exchange, biosorption, and electrochemical treatment^8,9^. The disadvantages of these methods are their insufficient selectivity, high operating costs, technical limitations when the pollutants concentrations are low, and the production of waste sludge containing heavy metal loads^10^.

To circumvent the disadvantages of conventional processes and/or make these processes more sustainable and effective, the use of alternative raw materials, such as lignocellulosic materials, are being incorporated into wastewater treatment processes. The valorization of agricultural waste biomass is more environmentally friendly because allows the use of various plant materials of easy access, low cost, abundant in nature, effective for low concentration of pollutants, minimum generation of toxic sludge, and easy regenerations for reuse^11,12^.

Biosorption is a treatment that allows the use of agro-industrial waste biomass, using lignocellulosic materials as biosorbents^9,13^. These materials have some characteristics that include: the existence of different functional groups such as alcohols, ketones, carboxylic acids, and phenols, that are present in the pores and/or on the surface of the adsorbent and affect their reactivity; high carbon content, which gives the material ideal hydrophobic characteristics for removal of oils and hydrocarbons; and controlled apparent density, to avoid its sedimentation at the bottom^14^.

The *Pachira aquatica* Aubl. (PA) is popularly known as *monguba, munguba, castanheira-do-Maranhão,* or *cacau selvagem*. A study by Correia et al.^15^ showed that biomass characterization has important properties for the biosorption process as hydroxyl groups, low moisture and ash content. *Monguba* is a fruit species that can be found from Southern Mexico to northern Brazil. Its fruits are similar to cocoa and the seeds have nutritional potential because they contain many proteins, lipids, and minerals, being widely studied as unconventional food plants (UFPs)^16^. The seed is also applicable as a biological controller due to the ethanolic extract that composes them, the oil obtained from the seeds can be used as an antioxidant and in biofuel production, and the leaves can be consumed as medicinal herbs^17,18^. Although PA has several applications, the peels of its fruits, which have woody characteristics and represent a large volume of the fruit (72.13%), have no defined application, they are treated as residues^15,19^.

Given the search for the development of sustainable materials for wastewater treatment and the use of waste. This work aimed to evaluate the applicability of a biosorbent produced from the biochar of the *Pachira aquatica* Aubl. fruit peel, which is considered an agricultural waste biomass, for the biosorption process aiming at the removal of Ni(II) and Cd(II) metal ions in batch and fixed bed column.

## Material and methods

### Biosorbent preparation

The biosorbent material used was obtained through the carbonization process of the peel of the fruit from *Paquira Aquatica* Aubl. (PA). The PA fruits were harvested in Natal, Rio Grande do Norte, Brazil. After being subjected to drying in an oven, the peels were ground in a mechanical grinder. Subsequently, they underwent a carbonization process at 450 °C for 4 h in a muffle furnace. The resulting biochar from the PA peel, referred to as PAB in this study, was sieved, selecting particle sizes between 0.3 and 1.0 mm. Later, this material was washed with deionized water, dried, and stored for future use as a biosorbent.

### Biosorbent characterization

The proximate analysis was conducted in accordance with ASTM (American Society for Testing and Materials) standards to determine the moisture content (ASTM E871-82), ash content (ASTM E1755-01), volatile matter content (ASTM E872-85), and fixed carbon content. The calculation for fixed carbon was determined using Eq. (1) below:

1 $${\text{Fixed}}\;\;{\text{carbon}}\;\;\% = {1}00 - \left( {{\text{moisture}} + {\text{ash}} + {\text{volatile}}\;\;{\text{matter}}} \right) \, \%$$

The PAB density was determined using a helium pycnometer performed in an Accupyc 1.330 equipment from Micrometics. The crystal structure of PAB was identified through X-ray diffraction (XRD) conducted on a D8 ADVANCE diffractometer from Bruker, utilizing CuKα radiation, at 1.000 W, data were collected from 5 to 70° (2θ) range. The morphology of PAB was analyzed through scanning electron microscopy (SEM) using an SSX-550 instrument from Shimadzu, operating at a voltage in 15 kV. The point of zero charges (pH_pzc_) determination involved mixing 0.15 g of PAB with 25.0 mL of 0.1 mol/L NaCl in multiple Erlenmeyer flasks. Initial pH (pH0) values of the NaCl solutions were adjusted from 3.0 to 11.0 by adding HCl and NaOH, then agitated at 150 rpm at room temperature for 24 h. Final pH values (pHf) were measured, and a pH_0_ versus pH_f_ graph was plotted. The zeta potential was measured using the ZetaPlus—Zeta potential analyzer from Brookhaven Instruments Corporation. PAB particles were suspended in an aqueous medium of pH 7.0 at room temperature for the analysis. Functional groups on the PAB surface were identified through the Boehm titration method. The adapted method by Moradi-Choghamarani et al.^20^ was employed for this characterization process.

### Statistical design of experiment

The design of the experiment aimed to optimize critical parameters in the biosorption process using PAB. A full factorial method was employed, assessing three independent experimental factors: (1) Temperature, (2) Biosorbent mass, and (3) Adsorbate concentration, with the response being the efficiency of metal removal (% removal, Eq. 1). he full factorial design (FFD) consisted of two levels for each factor (maximum and minimum), with three center points, resulting in a total of 11 experiments (Pk = 2^3^ + 3). The design of the experiment was conducted according to Table 1, To analyze the data, regression and graphical analysis were performed using STATISTICA 7.0 software.

**Table 1:** Experimental matrix of the full factorial design with three center points.

Design	Run order	Coded levels				Uncoded levels		
		Independent variables				Independent variables		
		T	m	C		T	m	C
						(°C)	(g)	(mg/L)
Factorial design	1	− 1	− 1	− 1	=	30	0.1	10
	2	+ 1	− 1	− 1	=	50	0.1	10
	3	− 1	+ 1	− 1	=	30	0.5	10
	4	+ 1	+ 1	− 1	=	50	0.5	10
	5	− 1	− 1	+ 1	=	30	0.1	70
	6	+ 1	− 1	+ 1	=	50	0.1	70
	7	− 1	+ 1	+ 1	=	30	0.5	70
	8	+ 1	+ 1	+ 1	=	50	0.5	70
Central design	9	0	0	0	=	40	0.3	40
	10	0	0	0	=	40	0.3	40
	11	0	0	0	=	40	0.3	40

### Biosorption tests

#### Bioadsorbent solution

For the biosorption tests, a stock solution containing the adsorbates was prepared by dissolving Ni(NO)_2_.6H_2_O (VETEC—97%) and Cd(NO_3_)2.6H_2_O (Dinâmica—99%) in deionized water. This process resulted in a synthetic multi-element solution containing 100 mg/L of Ni(II) and 100 mg/L of Cd(II). Further concentrations required for the experiment were derived through dilution from this initial solution.

#### Batch biosorption

Batch biosorption experiments were performed by employing 0.5 g of PAB within 25 mL of synthetic multi-element solutions in 125 mL Erlenmeyer flasks. These experiments were conducted in an agitation shaker (TE-420, Technal) under controlled and constant temperature conditions. Following the experiments, the samples were filtered through paper filters and were analyzed using an atomic absorption spectrophotometer (AAS), AA-6300 by Shimadzu, operating in flame mode and air acetylene burner. The percentage of metal removal (%R) was determined using Eq. (2), while Eq. (3) was employed to calculate its adsorption capacity (q, mg/g).

2 $$\% R=\frac{{C}_{0}-{C}_{t}}{{C}_{0}}. 100$$

3 $$q=\frac{\left({C}_{0}-{C}_{t}\right).V}{m}$$

where C_0_ represents the initial concentration (mg/L); C_f_ is the solution concentration at time t (mg/L); m is the mass of the biosorbent (g); and V is the volume of solution (L).

Blank controls were performed with the same experimental condition agitation, temperature, and concentration. These blank samples were crucial for monitoring potential analytical interferences throughout the experiments. Furthermore, the experiments were performed in triplicate to ensure statistical reliability and consistency in the obtained data.

Biosorption kinetics was conducted using 125 mL Erlenmeyer flasks containing 0.5 g of PAB and 25 mL of a synthetic multi-element solution of 70 mg/L. These flasks were securely sealed and placed within an incubator under consistent conditions: agitation set at 150 rpm, pH stabilized at 5, and a temperature maintained at 30 °C. Aliquots were taken at intervals of 5, 10, 15, 30, 45, 60, 90, 120, 150, 180, 240, 300, 360, 420 and 480 min. Kinetic models such as pseudo-first-order, pseudo-second-order and Intraparticle diffusion were adjusted to the experimental data using Origin® 6.0 software, to help understand the mechanisms involved in the process.

Biosorption isotherms were carried out using 125 mL Erlenmeyer flasks, each containing 0.5 g of PAB and 25 mL of synthetic multi-element solutions with varying concentrations: 10, 20, 30, 40, 50, 60, 70, 80, 90, and 100 mg/L. These flasks underwent continuous agitation at 150 rpm, at temperature of 30 °C and pH 5 during 180 min. The experimental data was fitted using the Langmuir (non-linear) and Freundlich (non-linear) models. These models were adjusted using Origin® 6.0 software for the analysis and fitting process.

The biosorption thermodynamic analysis was conducted for 180 min using 0.5 g of PAB in 25 mL of synthetic multi-element solutions, the concentrations ranged from 20 to 100 mg/L. The experiments process maintained a constant agitation at 150 rpm and pH 5, and the temperature was systematically adjusted across a range of 30, 40, 50, and 60 °C.

The results obtained in the adsorption experiments were analyzed based on the Langmuir and Freundlich isotherm models for equilibrium data, while the kinetic data were studied according to the models of Pseudo first-order, Pseudo second-order, and intraparticle diffusion. Table S.1 (Supplementary material) presents the models and equations used to evaluate batch biosorption data.

#### Fixed-bed column biosorption

Column experiments were carried out in a glass column with an internal diameter of 1.5 cm and a length of 23.5 cm. PAB was packed inside the column, and a layer of glass wool was fitted at the bottom and at the top of the column to support the packing. The synthetic multi-element solution was pumped into the column in descending flow using a peristaltic from Technal.

In the fixed-bed column biosorption tests, variations were made in both the height of the fixed-bed biosorption and the initial concentration of the adsorbate, resulting in three distinct tests. Column A, utilized a fixed-bed biosorption height of 9.0 cm and an adsorbate initial concentration of 100 mg/L. In the second test, Column B, while maintaining consistent adsorbate initial concentrations, the fixed-bed biosorption height was increased to 12.0 cm. Finally, Column C, a fixed-bed biosorption height of 12.0 cm was paired with adsorbate initial concentrations set at 45 mg/L. All three experiments were conducted at an identical flow rate (3 mL/min) and were performed in triplicate. The operational parameters of the column were calculated according to the equations described in Table S.2 (Supplementary material).

## Results and discussions

### Bioadsorbent characterization

**Figure 1:**
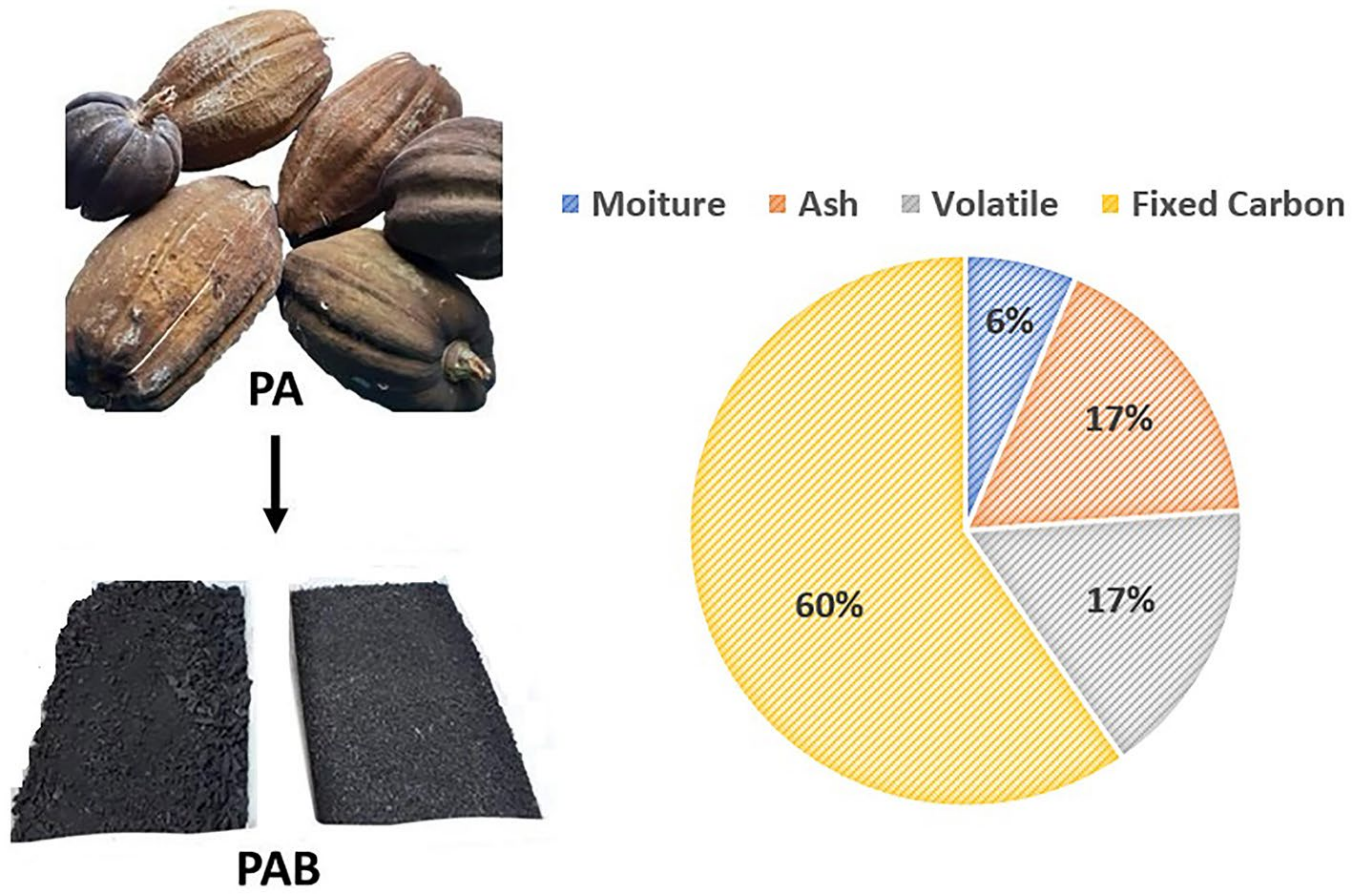
Proximate analysis results of PAB.

Proximate analysis of PAB presented 6.55% of moisture level, 59.61% of fixed carbon, 17.28% of ash content and 16.55% of volatile content, as shown in Fig. 1. The advantageous nature of the biosorption process is underscored by the notably low PAB moisture level of 6.55%. Substances exhibiting heightened moisture content frequently encounter a diminished adsorptive capacity due to pore occupation by water molecules, thereby impeding the adsorption process for alternative adsorbates.

The largest component of the PAB was fixed carbon at 59.61%, a result associated with the decomposition of the lignocellulosic components present in the material. This substantial concentration of fixed carbon content is favorable to the adsorption process, since the porous nature and molecular structure of carbon allows the formation of bonds with other molecules, thereby significantly amplifying the material's capacity to adsorb chemical substances.

The ash content of 17.28% is inherently associated with the mineral elements present, presumably originating from the chemical breakdown of PAB during biomass carbonization. Simultaneously, biomass volatilization led to a volatile content of 16.55%. Elevated levels of ash and volatiles may influence the adsorption process, contingent upon the quantity of silica, heavy metals, and volatile substances in competition with other active adsorption sites^21^.

The density and volume of the PAB were 1.7094 g/cm^3^ and 0.4787 cm^3^, respectively. According to Dune et al.^22^, that higher carbon densities provide an adequate porous structure, in addition to maintaining the physical and chemical integrity of the bioadsorbent during the adsorption process. The density of PAB in relation to other biosorbents, such as those sourced from coconut shells (ranging between 0.49 and 0.68 g/cm^3^), it becomes evident that PAB emerges as a superior option due to its notably higher density. Additionally, the density exhibited by PAB aligns closely with that of commercially available activated carbons (typically 2.0–2.1 g/cm^3^).

The analysis by XRD depicted in Fig. S.1 (Supplementary material) showed that the PAB presented a primordially amorphous structure with disordered plane characteristics of biochar, in which the absence of crystallinity is associated with the removal of holocellulose in the carbonization process. Amina Soudani et al.^23^ conducted a study on the crystalline structure of biochar, revealing two distinct peaks. One peak was associated with a disordered structure of aromatic carbon, while the other peak indicated the substantial presence of amorphous carbon featuring a wide range of pore size distribution. These properties notably improve the adsorption capacity of coals.

**Figure 2:**
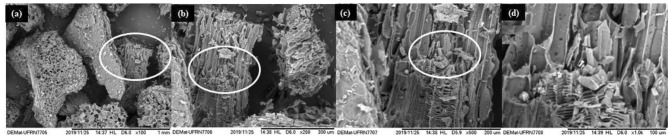
SEM images of the PAB in magnification of (**a**) 100, (**b**) 250, (**c**) 500 E (**d**) 1000 times.

The PAB micrographs (Fig. 2) allow the observation of pores of different shapes and sizes and the rough surface with the presence of porous channels from the plant cell wall structure of the precursor biomass. The formation of pores on the surface of biochar and the expansion of the diameter of existing pores are attributed to the carbonization process. In the image with an increase of 1000x, it is possible to notice more clearly the irregularity of the surface, as well as the difference between the size of the pore channels.

It can be inferred that the surface of the PAB is porous, irregular, and heterogeneous, these characteristics are favorable for the biosorption process of heavy metals in an aqueous solution, since the adsorbed components are concentrated on the external surface and the larger this external surface per unit solid mass, the more favorable the process will be.

**Figure 3:**
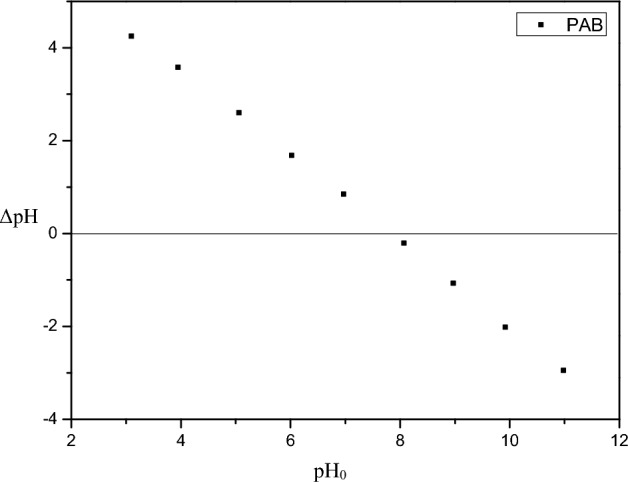
pH at point of zero charges (pH_pcz_) of the PAB.

The point of zero charges pH (Fig. 3) for PAB was 7.6, that is, it is within the neutrality range (6.0–7.6), being able to perform cations and anions biosorption. It is known that when the pH of the solution is lower than the pH_pcz_, the surface of the material becomes positive, therefore, it repels positively charged ions. And when the pH of the solution is higher than pH_pcz_, the surface of the material has a negative charge, so it attracts positively charged ions^24^. For that reason, the PAB favors the cations biosorption in wastewater with a pH greater than 7.6, such as textile industry and pig slaughterhouse wastewater^25,26^.

The mean value for the zeta potential of PAB was − 56.94 mV. The strongly negative result indicates that the PAB surface has an affinity with cations, in which there will be an increase in the electrostatic attraction force between the biosorbent and the adsorbates Cd(II) and Ni(II)^27^. The negatives charges on the surface of the material are related to the types of functional groups that compose it, and these groups favor the cationic biosorption process as they keep the adsorbate inside the pore, assisting in the formation of the complexes that increase the biosorption capacity of the material^4^.

The Boehm titration was observed presence of carboxylic groups (1.03 mmol/g), phenolic groups (0.57 mmol/g), and lactonic groups (0.57 mmol/g), which are functional groups capable of efficiently connecting with heavy metal, being favorable to the biosorption process. The presence of these groups was also observed on the PAB surface in the FTIR performed by Júnior et al.^28^, from the identification of bands 3725–3792 cm^−1^ related to axial deformations in the O–H group. Besides that, there were observed peaks at 3000–2700 cm^−1^, 1800–1100 cm^−1^, and 900–500 cm^−1^ related to the stretching of the C–H group, the vibration of C=C bond, and the deformation of C–H bond, respectively. These groups serve as potential sites for an efficient biosorption of cations confirmed by Patra et al.^29^ who reported major changes in the intensities of band peaks before and after cation biosorption.

The basic surface and the formations of bands 1700–1600 cm^−1^ referring to the elongation vibration of C=O, identified in the FTIR performed by Júnior et al.^28^, are related to the presence of basic oxygenated functional groups, such as hydroxyl, carbonyl, and carboxyl groups. These groups are recognized as one of the main contributors to the arrangement between heavy metals and adsorbent surfaces, acting as active sites^30^. Moreover, the existence of negatively charged functional groups on the PAB surface indicates that compounds with a positive charge (cations) will be more easily absorbed by this material due to the electrostatic interaction that will occur between cations and anions^31^.

### Statistical design of experiment

The Pareto chart, in Fig. S.2 (Supplementary material), shows in decreasing order of relevance, the effects of each factor and its interactions in the response variable. The factors related to the interaction of temperature with adsorbate concentration for Ni(II) removal and interactions of temperature and biosorbent mass for Cd (II) removal were insignificant to the process, indicating that for Ni(II) the adsorbate concentration and Cd(II), the biosorbent mass is independent of the temperature variation of the process. The adsorbate concentration was the factor that most affected the process, presenting negative effects. Therefore, the reduction of adsorbate concentration in the process will lead to greater removals of metal ions due to the higher amount of available binding sites.

**Figure 4:**
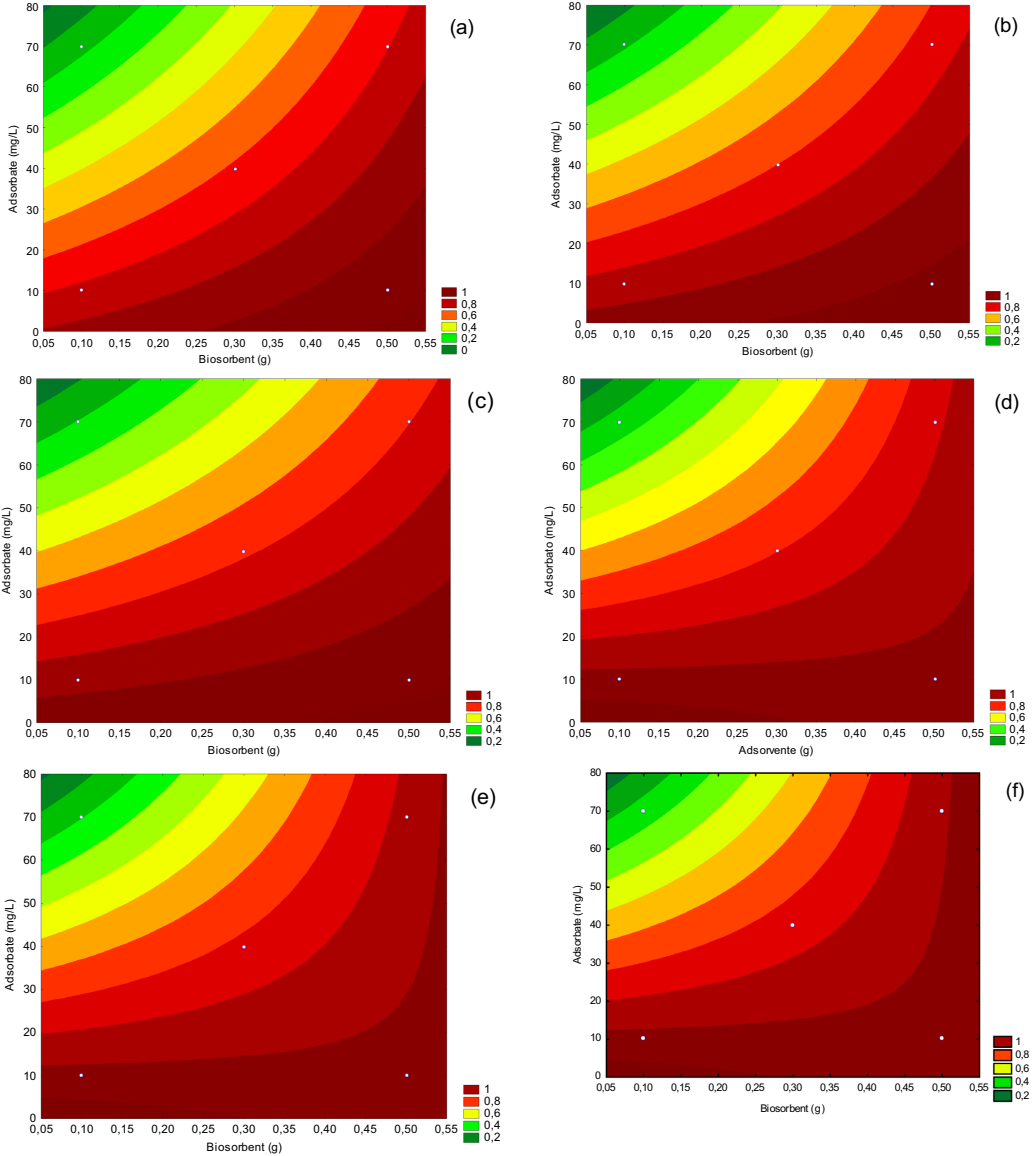
Contour plot for removal efficiency of Ni(II) in 3 temperatures: (**a**) T = 30 °C, (**b**) T = 40 °C e (**c**) T = 50 °C and Cd(II) in three temperatures: (**d**) T = 30 °C, (**e**) T = 40 °C e (**f**) T = 50 °C.

From the contour plot (Fig. 4) is possible to observe that for all temperatures, the red band (which indicates removal > 80%) presented similar sizes in that the 100% removal interval, slightly large for T = 50 °C. Among the beneficial effects of temperature increase is the decrease in the solution viscosity, which causes an increase in the adsorbate diffusion rate in the limit layer of the pores of the adsorbent. The possibility of pore clearance, allowing the penetration of larger adsorbate molecules; and the increase in the degree of agitation of adsorbate molecules, which allows greater contact with the biosorbent and promotes greater biosorption^39^.

Comparing the surfaces of both ions, under the same temperature, it can be inferred that the biosorbent is more efficient for Cd(II) removal, since the areas referring to the removal range of 80–100% were higher for this ion, at all temperatures. Similar results were observed by Nascimento et al.^40^ in their tests for biosorption of Ni(II) and Cd(II), besides Fe(II), in charcoal produced from the peels of *Pachira aquatica* Aubl. It is also observed that, for any fixed metal concentration, the increase in biosorbent mass generates an increase in the metal ion removal, this behavior was expected since the increase in the biosorbent amount increases the number of sites available for biosorption^24^.

A linear mathematical model was obtained that correlates the independent variables with the removal of Ni(II) and Cd(II) through the biosorption process. The models are described by Eq. (4) and (5) for Ni(II) and Cd(II), respectively, and these were evaluated by ANOVA described in Table S.3 (Supplementary material) in which it can be affirmed that the calculated linear model is statistically significant for the study of Ni(II) and Cd(II) removal through the biosorption process using the PAB. In which x_T,_ x_mb_, and x_ca_, refer to the values of (°C), biosorbent mass (g), and adsorbate concentration (mg/L), respectively:

4 $$\eta_{{{\text{Ni}}}}^{{{2} + }} = 0.{37}0{633} + 0.0{\text{13825x}}_{{\text{T}}} + 0.{93152}0{\text{x}}_{{{\text{mb}}}} - 0.0{1214}0{\text{x}}_{{{\text{ca}}}} - 0.0{\text{16149x}}_{{\text{T}}} {\text{x}}_{{{\text{mb}}}} + 0.0{\text{15882 x}}_{{{\text{mb}}}} {\text{x}}_{{{\text{ca}}}}$$

5 $$\eta_{{{\text{Cd}}}}^{{{2} + }} = {1}.{221}0{62} - 0.0{\text{18337 x}}_{{{\text{ca}}}} + 0.0000{\text{85 x}}_{{\text{T}}} {\text{x}}_{{{\text{ca}}}} + 0.0{\text{26567 x}}_{{{\text{mb}}}} {\text{x}}_{{{\text{ca}}}}$$

### Biosorption tests

#### Biosorption kinects

**Figure 5:**
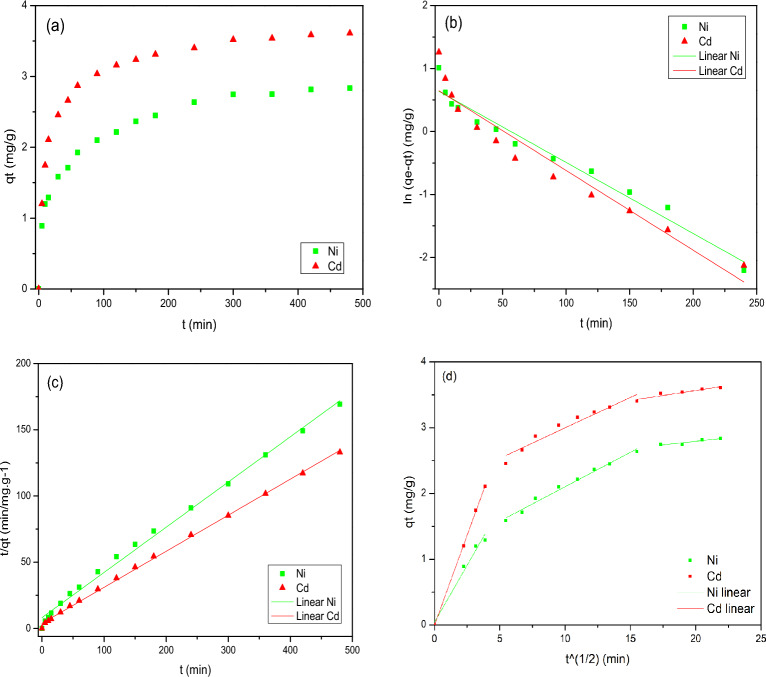
(**a**) Biosorption Kinetic of the Ni(II) e Cd(II) metal ions using the PAB biosorbent and Kinetics models of (**b**) Pseudo first-order, (**c**) Pseudo second-order and (**d**) Intraparticle diffusion linear and non-linear.

The results obtained for the kinetic biosorption, Fig. 5a, it was possible to observe that the biosorption process was slow and occurred continuously, taking about 300 min to reach equilibrium. At this point, the removal of the ions was 81% for Ni(II) and 96% for Cd(II), with the biosorption capacity (q_e_) of 2.7 mg/g and 3.5 mg/g of Ni(II) and Cd(II), respectively. The difference in metal ion removal rates can be attributed to the ion exchange capacity of the metal with the functional groups on the PAB surface.

To investigate the rate and the controlling mechanism of the biosorption process, the pseudo first-order model, Fig. 5b, pseudo second-order model, Fig. 5c, and intraparticle diffusion linear and non-linear model, Fig. 5d, were fitted to the experimental data. The parameters for each model are shown in Table 2.

**Table 2:** Adjustment parameters obtained from Pseudo first-order, Pseudo second-order, and Intraparticle diffusion models for the biosorption of the Ni(II) and Cd(II) metal ions.

Kinetic model		Parameters	Ni(II)	Cd(II)
Pseudo first-order		q_e_ (mg/g)	1.89	1.91
		K_1_ (min^−1^)	− 0.0113	− 0.0127
		R^2^	0.931	0.968
Pseudo second-order		q_e_ (mg/g)	2.93	3.69
		K_2_ (min/g.mg^−1^)	0.0143	0.0192
		R^2^	0.995	0.999
Intraparticle diffusion (Linear)				
		C (mg/g)	Kd*	R^2^
Ni	I	0.0422	0.3463	0.9720
	II	1.0561	0.1047	0.9932
	III	2.3622	0.0216	0.8116
Cd	I	0.000	0.5465	0.9996
	II	2.0735	0.0925	0.9284
	III	3.1636	0.0203	0.9412

*(min/g.mg^1/2^).

Comparing the statistical coefficients obtained of the adjustments, it can be inferred that all the adsorption systems conformed to the pseudo second-order model, as evidenced by the R^2^ parameters nearing unity. In addition to the R^2^ values, assessing the suitability of the proposed models also entails comparing the calculated adsorption capacity values (qe) obtained through with the experimental values (qe, exp) observed at equilibrium time (240 min).

Since, q_e_ is closer to the experimentally obtained value (q_exp_), the pseudo second order model can be considered as the appropriate model to describe the kinetic adsorption of Ni(II) and Cd(II) ions on PAB. According to the data presented in Table 2, there is the following: the adsorption capacity of experimental Ni(II) was 2.7 mg/g and that predicted in the second-order model was 2.93, for Cd(II), the experimental value was 3.5 and the value predicted by the model was 3.69, therefore, the pseudo second-order model was the one that provided the best fit since it performed a data prediction closer to the experimental data.

According to Pal and Pal^32^, if the biosorption process follows the pseudo second-order model, the removal is assumed due to the physical–chemical interaction between the adsorbate and biosorbent, which involves the donation or sharing of electrons, from the covalent or ionic bonding forces. This model is recommended for a process that occurs in a longer period of biosorption, which occurred for biosorption with PAB^24^. Moreover, the molecules in this type of biosorption are not attracted to the entire surface of the solid, but specifically by activated sites to initially form a monolayer, with the possibility of the formation of another layer by physisorption^33^. The continuous behavior of kinetic curves suggests the occurrence of monolayer biosorption, behavior characteristic to the chemisorption processes.

Applying the nonlinear intraparticle diffusion model, as represented in Fig. 5d, it becomes possible to verify a significant improvement in the fit compared to the application of the same model, both linear and nonlinear, evidenced by an increase in the value of R^2^. However, this increase has not yet reached ≥ 0.99. In this study, it was observed that at the beginning of the process, there is diffusion of metal ions towards the external surface of the adsorbent, resulting in a faster adsorption rate, especially for Ni(II). The second stage may be associated with diffusion in the mesopores, due to the heterogeneity present on the surface of the bioadsorbent. While the third stage, with a smoother and practically linear slope, is related to diffusion in the micropores. This suggests that intraparticle diffusion of ions in the micropores was the rate-limiting stage in the biosorption process of Ni(II) and Cd(II) on PAB, especially at longer contact times and higher concentrations.

#### Biosorption isotherms

**Figure 6:**
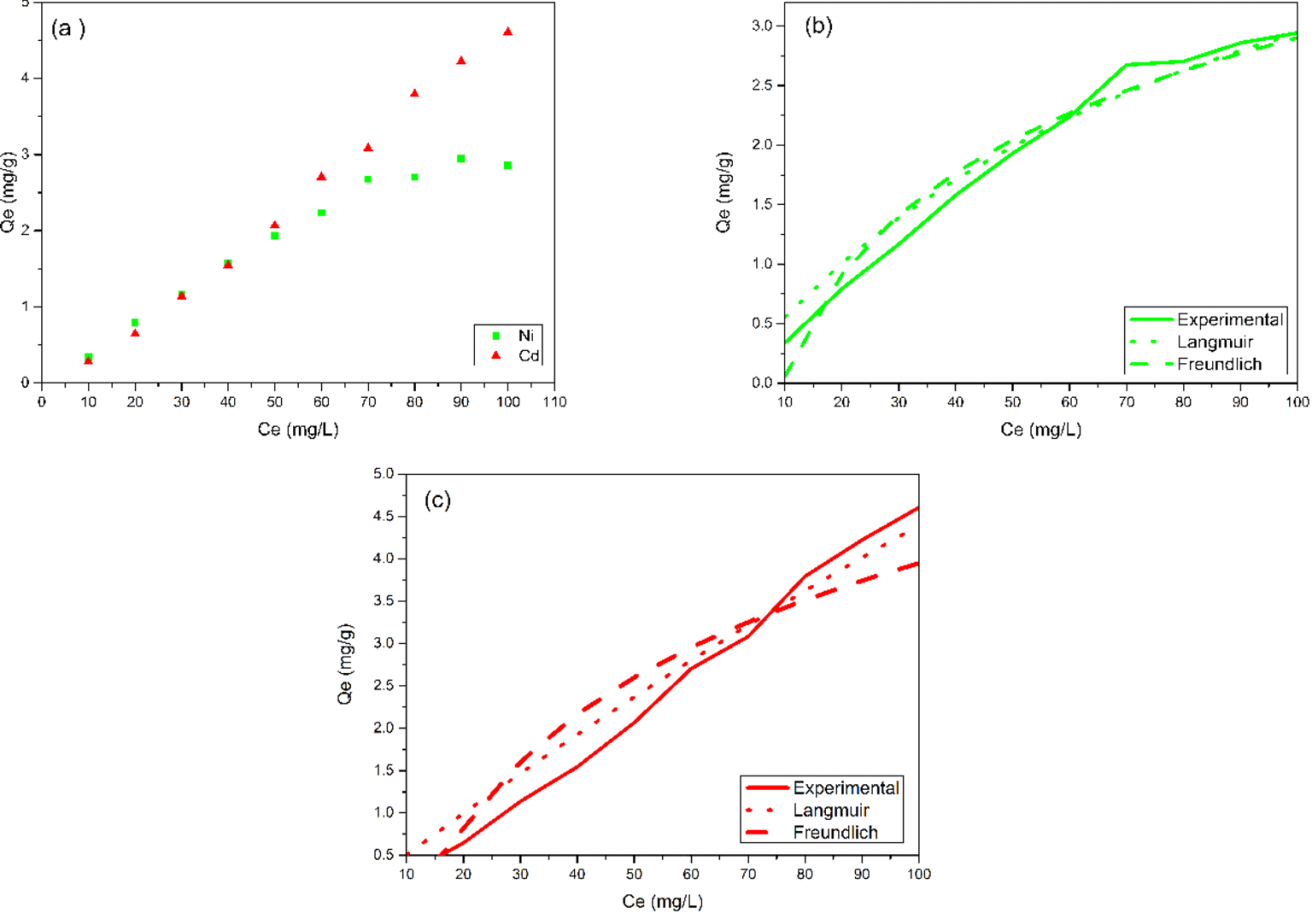
(**a**) Biosorption isotherms of the Ni(II) e Cd(II) metal ions using the PAB biosorbent and Langmuir and Freundlich models for the biosorption of the (**b**) Ni(II) and (**c**) Cd(II) metal ion.

The distribution of Ni(II) and Cd(II) ions by PAB over the entire concentration range investigated (10–100 mg/L) was analyzed using the Langmuir and Freundlich isotherm models in individual systems. The experimental data, along with the isotherms obtained through nonlinear analysis, depicted in Fig. 6a. The determined parameters and their corresponding values for these isotherms are detailed in Table 3.

**Table 3:** Adjustment parameters obtained from Langmuir and Freundlich models for the biosorption of the Ni(II) and Cd(II) metal ions.

Isotherm model	Parameters	Ni(II)	Cd(II)
Langmuir	q_máx_ (mg/g)	5.65	30.44
	K_L_ (L/g)	0.0109	0.0017
	R_L_	0.48–0.90	0.86–0.98
	R^2^	0.922	0.885
Freundlich	N	0.35	0.22
	1/n	2.84	4.48
	K_f_*	0.00160	0.00001
	R^2^	0.9627	0.8837

*(mg.g^−1^)(L.mg^−1^)^1/n^.

The biosorption isotherm profile, Fig. 6a, indicates that there is an increase in the biosorption capacity with the increase of adsorbate concentration, in which, the maximum concentration used (100 mg/L) for Cd(II) provided greater biosorption capacity. For Ni(II) was until the concentration of 70 mg/L, because after this value the biosorption capacity becomes practically constant. The concentration gradient, which becomes larger as the adsorbate concentration increases, promoted number of collisions between the biosorbent and the ions, causing a faster mass transport, due to an increase in the diffusion coefficient or mass transfer coefficient, consequently, the biosorption capacity will be higher^34,35^.

The graphs present in Fig. 6b,c revealed a remarkable conformity of the experimental data with the linearized equation of the Langmuir isotherm across all variations of Cd(II) and Ni(II) ion concentrations examined. As shown in Table 3, the R^2^ values found were 0.922 for Ni(II) and 0.885 for Cd(II), indicating that the Langmuir model better describes the affinity between the biosorbent surface and the adsorbate than the Freundlich model (R^2^ = 0.8837–0.9627). Langmuir model suggests that the adsorption occurred in monolayer and homogeneous. The maximum theoretical biosorption capacities given from Langmuir model (q_max_) were 5,65 and 30,44 for Ni(II) and Cd(II), respectively.

According to Liu and Lian^36^, although Ni(II) has a greater affinity for the biosorbent surface due to its greater electronegativity, is proven by its higher value of *n* which represents the interaction force biosorbent/adsorbate, the largest ionic radius of Cd(II) can prevent Ni(II) access to the pores of the biosorbent. Thus, the ionic radius is better applicable as an indicator of affinity between biosorbent surface and adsorbate comparing mononelementary systems where there are no ions dispute. From the evaluation of the parameters obtained for each model, it is possible to verify, mainly from the order of biosorption capacity, then Langmuir model promoted a better description of the experimental data.

In agreement with the Langmuir isotherm model, biosorption occurs in monolayer and metal uptake occurs on a homogeneous surface^9^. The monolayer biosorption suggested by the Langmuir model confirms the results obtained for the biosorption kinetics described by the pseudo second-order model, indicating the occurrence of chemisorption.

#### Biosorption thermodynamics

The thermodynamic parameters evaluated, in Table 4, demonstrate that the biosorption occurred in a way favorable and spontaneous (ΔG < 0), endothermic (∆*H* > 0), and with increased randomness of the system during sorption (∆*S* > 0). Negative values for ΔG accompanied by positive values for ΔS indicate that the biosorbent has an affinity for the adsorbate.

**Table 4:** Thermodynamic parameters for the biosorption of the Ni(II) and Cd(II) metal ions.

Ions	Temperature	ΔG	ΔH	ΔS	R^2^
	°C	kJ/mol	kJ/mol	kJ/mol.K	
Ni(II)	30	−59.02	54.88	0.014	0.81
	40	−59.15			
	50	−59.29			
	60	−59.42			
Cd(II)	30	−124.69	119.83	0.034	0.72
	40	−124.85			
	50	−125.01			
	60	−125.17			

The endothermic process explains the positive influence of temperature increase in the biosorption process using the PAB. In addition, the high values of ∆*H* revealed that chemisorption has occurred, as they are within the range 20.9—418.4 kJ/mol taken as the heat range of the chemisorption^37^. Ogbodu et al.^38^ reported the occurrence of chemisorption in the biosorption process of Ni(II) and Cd(II) using Parkia biglobosa biomass in which the biosorption capacity followed the order indicated in this study [Cd(II) > Ni(II)].

#### Fixed-bed column biosorption

**Figure 7:**
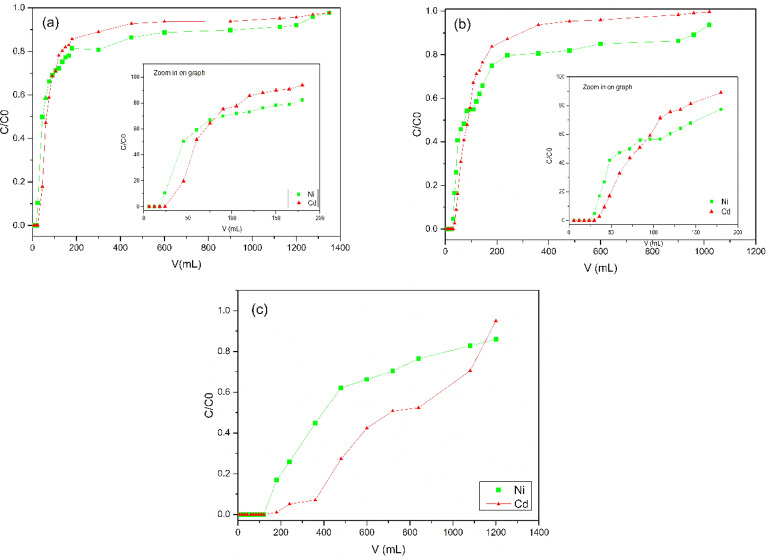
(**a**) Column A with 9 cm and concentration of 100 mg/L, (**b**) Column B with 12 cm and concentration of 100 mg/L, and (**c**) column C with 12 cm and concentration of 45 mg/L.

The breakthrough of columns A, B, and C (Fig. 7) for Ni(II) and Cd(II) presents a slight asymmetrical S-shaped curve, characteristic of the ideal systems. Through the operational parameters of the column (Table 5) it is seen that the time to reach the exhaustion point (t_x_) and the rupture point (t_b_) was higher for column C, with consequently higher volumes of exhaustion (V_x_) and rupture (V_b_). This result is related to the bed depth increased from 9 to 12 cm, resulting in an increased amount of available binding sites for the column biosorption and increased contact time^34,35^. In addition, the reduction of adsorbate concentration from 100 gm/L to 45 mg/L decreased the driving forces for mass transfer, making the binding sites saturate more slowly^41^.

**Table 5:** Operational parameters in a fixed-bed column for Ni(II) and Cd(II) biosorption by PAB.

Parameter	Column A		Column B		Column C	
	Ni(II)	Cd(II)	Ni(II)	Cd(II)	Ni(II)	Cd(II)
C/C_0_ Máx	0.98	0.98	0.94	0.99	0.86	0.95
t_b_ (min)	6.0	8.0	10.0	12.0	60	80
t_x_ (min)	300.0	60.0	320.0	80.0	400	360
V_b_ (mg/L)	18.0	24.0	30.0	36.0	180	240
V_x_ (mg/L)	900.0	180.0	960.0	240.0	1200	1080
δ (cm)	8.1	7.4	10.5	5.5	7.8	8.4
%sat	23.9	26.7	31.0	59.5	47.8	49.3

The C/C_0_ ratio of both ions for all columns did not reach the unit value, then the total saturation of the biosorbent bed was not reached, but it came very close to this result. This can be confirmed by the percentage of positive saturation (%sat) of the columns.

The C/C0 ratio of both ions for all columns did not reach the unit value, indicating that the complete saturation of the adsorbent bed was not achieved. However, it came very close to this result. This can be confirmed by the positive saturation percentage (%sat) of the columns.

The lengths of the mass transfer zone—MTZ (δ) obtained for the metal ions in the three columns were shorter than the length of the biosorbent bed. Comparing the results obtained, it can be seen that the shorter lengths of MTZ were achieved with column C for Ni(II) and with column B for Cd(II). The smaller the MTZ, the closer the system is to ideality. In view of this, the results show that there is competition between Ni(II) and Cd(II) ions for active sites, where, Cd(II) is favored, behavior recorded in different studies^42^. However in lower concentrations, there is less competition due to greater availability of active sites, which allows the biosorption of higher amounts of Ni(II).

This study underscores the significance of operational parameters and their influence on biosorption dynamics, shedding light on competitive interactions between metal ions. The findings offer valuable insights into optimizing biosorption processes for improved efficiency in treating metal-contaminated systems, emphasizing the importance of further research in this area to enhance our understanding and application of biosorption technologies.

### Mechanisms of Ni(II) and Cd(II) biosorption by PAB

**Figure 8:**
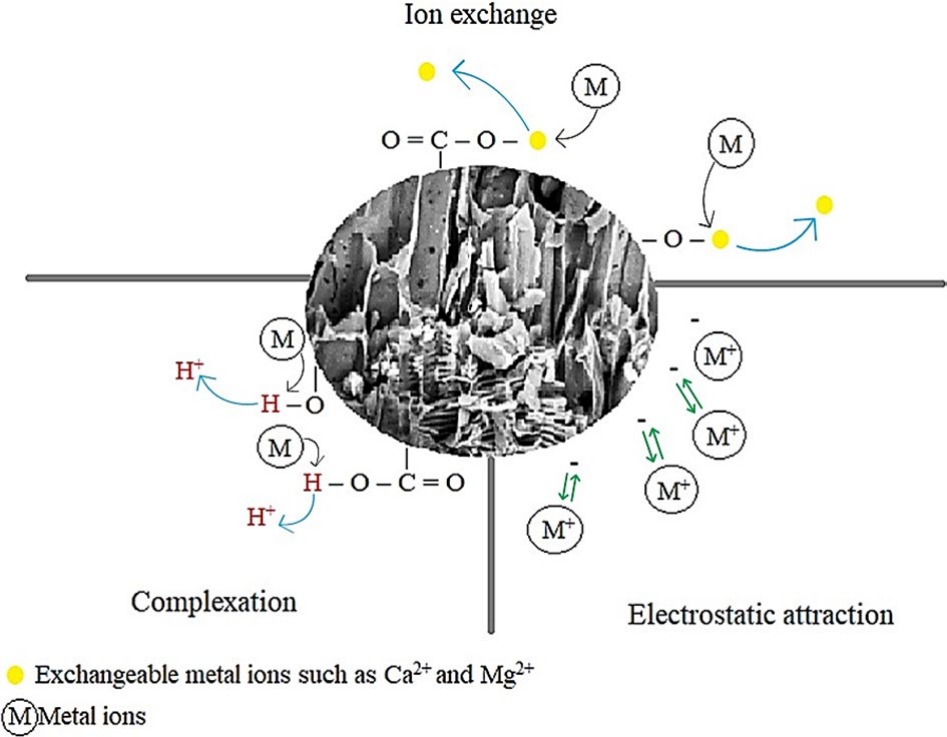
Illustration of heavy metal biosorption mechanisms on PAB.

The results obtained in all experiments indicate that the PAB has good biosorption capacity for Ni(II) and Cd(II). Three biosorption mechanisms were proposed for Ni(II) and Cd(II) removal: Ion exchange, complexation, and electrostatic interaction, as shown in Fig. 8.

The Boehm titration test and FTIR showed the presence of acid groups –OH and –COOH on the surface of the PAB. The presence of these groups indicates the occurrence of cation biosorption from the complexion mechanism^30^. The presence of these groups indicates the occurrence of cation biosorption from the complexation mechanism. Such effects highlight the surface binding of metals to carboxyl (–COOH, O–C), carbonyl (C=O), hydroxyl (–OH), or amine (–NH) groups present in the biomass. This happens through electrostatic interactions or π–π interactions between aromatic groups of the biomass and the metals.

The presence of basic groups in predominance and groups with a negative surface, point to the occurrence of electrostatic interaction and ion exchange between the PAB surface and cations, whose ion exchange occurs between cations and light bivalent metals, such as Ca(II) and Mg(II), found on the surface of biosorbents from lignocellulosic waste materials^43^.

According to Chao and Chang^44^, if the ion exchange is the primary mechanism of biosorption, the biosorption capacities of the heavy metals must be proportional to the ionic radius, in which, Ni(II) has a relatively higher potential to form complexes and the Cd(II) generates higher biosorption amounts through ion exchange. In the present study, the order of biosorption capacities was proportional to the ionic radius and the highest biosorption capacity was for Cd(II) indicating ion exchange as the main mechanism.

## Conclusions

According to the present results, the PAB has the potential as a biosorbent of Ni(II) and Cd(II) due to its characteristics of high porosity, presence of functional groups, and bond groups, which favor the biosorption, and negative charge of its surface, that promotes electrostatic attraction and ion exchange with cations. The results of the batch biosorption tests showed that the process occurred slowly, continuously, and in monolayers. The interaction between the PAB and biosorbent occurred via chemisorption, and the main mechanisms involved in the process were ion exchange, complexation, and electrostatic attraction. The column biosorption showed that the biosorption capacity was higher for the Cd(II) than the Ni(II), but using lower initial concentrations (45 mg/L), higher amounts of Ni(II) were removed indicating that there was competition for binding sites, in which Cd(II) was favored due to its higher ionic radius. Thus, it is concluded that the PAB has the potential to remove heavy metals in the process of biosorption in batch process and fixed bed columns. There is also the possibility of applying the PAB as part of a combined process and/or tertiary treatment. This characteristic presents promising applications in wastewater treatment, air purification, and environmental remediation. Harnessing the inherent properties of fixed carbon within PAB holds potential for innovative solutions across industries, emphasizing its crucial role in enhancing adsorption processes for diverse applications.

### Supplementary Information

Supplementary Information.

## Data Availability

Experimental data can be available from corresponding authors on reasonable request.
