# Supplementary material for: Biosorption of nickel and cadmium using Pachira aquatica Aubl. peel biochar
Source: Sci Rep. 2024 Mar 1;14:5086. doi: 10.1038/s41598-024-54442-w (PMC10907595; doi:10.1038/s41598-024-54442-w)
Supplement: Supplementary file 1 — Supplementary Information. [file 41598_2024_54442_MOESM1_ESM.docx]

**Supplementary material**

Table S.1. Models used for the evaluation of adsorption of Ni(II) and Cd(II) ions in PAB.

| **Isotherm** | | |
| --- | --- | --- |
| **Model** | **Equation** | **Parameters** |
| Langmuir | $q_{eq}=\frac{q_{max}K_{L}C_{eq}}{1+K_{L}C_{eq}}$ | q_eq_ (mg/g): Sorption capacity at equilibrium  q_max_ (mg/g): Maximum sorption capacity |
|  | $R_{L}=\frac{1}{(1+K_{L}C_{o})}$ | K_L_ (L/mg): Langmuir’s constant  C_eq_ (mg/L): concentration at equilibrium R_L_: separation factor  C_o_ (mg/L): higher initial concentration |
| Freundlich | $q_{eq}=K_{F}C_{eq}^{\frac{1}{n}}$ | K_F_ (mg/g)(L/mg)^1/n^: Freundlich’s constant  n: heterogeneity factor |
| **Kinetic** | | |
| **Model** | **Equation** | **Parameters** |
| Pseudo first-order | $lnln \left( q_{eq}-q_{t} \right) =lnln q_{eq}- k_{1}t$ | q_t_ (mg/g): sorption capacity at time t  k_1_ (1/min): pseudo first-order’s constant rate |
| Pseudo second-order | $\frac{1}{q_{t}}=\frac{1}{k_{2}q_{eq}^{2}}+\frac{1}{q_{eq}}t$ | k_2_ (1/min): pseudo second-order’s constant rate |
| Intraparticle diffusion | $q_{t}=k_{d}t^{0,5}+C$ | k_d_ (mg/min^1/2g^): instraparticle diffusion’s parameter  C (mg/g): intercept |
| **Thermodinamyc** | | |
| **Equation** |  | **Parameters** |
| $\Delta G^{^{\circ}}=\Delta H^{^{\circ}}-T\Delta S^{^{\circ}}$ | Enthalpy change ($\Delta H^{^{\circ}}$) and entropy change ($\Delta S^{^{\circ}}$) can be obtained from the plot of log$(\frac{q_{e}}{c_{e}})$ vs. 1/T. | ΔG°: Gibbs free energy  T: Temperature (K)  $q_{e}=$ amount of metal adsorbed by unit mass of adsorbent (mg/g)  $c_{e}=$ Equilibrium metal concentration (mg/L) |

Table S.2. Operational parameters for column biosorption studies

| **Column adsorption** | | |
| --- | --- | --- |
| **Operational parameter** | **Equation/Description** | **Parameters** |
| C/C_0_ Máx | - | C = concentração final (mg/L);  C_0_ = Initial concentration (mg/L) |
| t_b_ (min) | Time at C/C_0_ = 0,05 | t_b_ = Breakthrough time (min) |
| t_x_ (min) | Time at C/C_0_ = 0,95 | t_x_ = Exhaust time (min) |
| V_b_ (mg/L) | Volume at C/C_0_ = 0,05 | V_b_ = Breakthrough volume (mg/L) |
| V_x_ (mg/L) | Volume at C/C_0_ = 0,95 | V_x_ = Exhaust volume (mg/L) |
| δ (cm) | $\delta=\left( 1-\frac{q_{u}}{q_{t}} \right)*h$ | δ= Height of ZTM;  q_u_ = Amount of metal removed until the breakthrough point (mg);  q_t_ = Total amount of metal removed (mg);  h = Height of fixed bed adsorption (cm) |
| %sat | %S = $\left[ 1+\left( \frac{\delta\left( f-1 \right)}{h} \right) \right]*100$ | %S = Column saturation percentage;  δ = Height of ZTM (cm);  *f* = Adsorbent capacity fractional in the adsorption zone to continue removing solute after the breakthrough point;  h = Height of fixed bed adsorption (cm). |

Table S.3. Analysis of variance for removed Ni(II) and Cd(II) using PAB

| **Metal ion** |  | **Source** | **Sum of squares (SS)** | **Degrees of freedom**  **(df)** | **Mean square (MS)** | **F** | **F_tab_** |
| --- | --- | --- | --- | --- | --- | --- | --- |
| **Ni(II)** |  | Regression (R) | 0,762858 | 3 | 0,254286 | 14,91 | 4,35 |
|  |  | **Residual (r)** | **0,119312** | **7** | **0,017045** |  |  |
|  |  | Lack of fit | 0,038192 | 2 | 0,019096 |  |  |
|  |  | **Pure error** | **0,000072** | **2** | **0,000036** |  |  |
|  |  | **Total SS** | **0,882170** | **10** |  |  |  |
| **Cd(II)** |  | Regression (R) | 0,557341 | 3 | 0,185780 | 5,48 | 4,35 |
|  |  | **Residual (r)** | **0,237146** | **7** | **0,033878** |  |  |
|  |  | Lack of fit | 0,028471 | 2 | 0,014235 |  |  |
|  |  | **Pure error** | **0,000176** | **2** | **0,000088** |  |  |
|  |  | **Total SS** | **0,794487** | **10** |  |  |  |


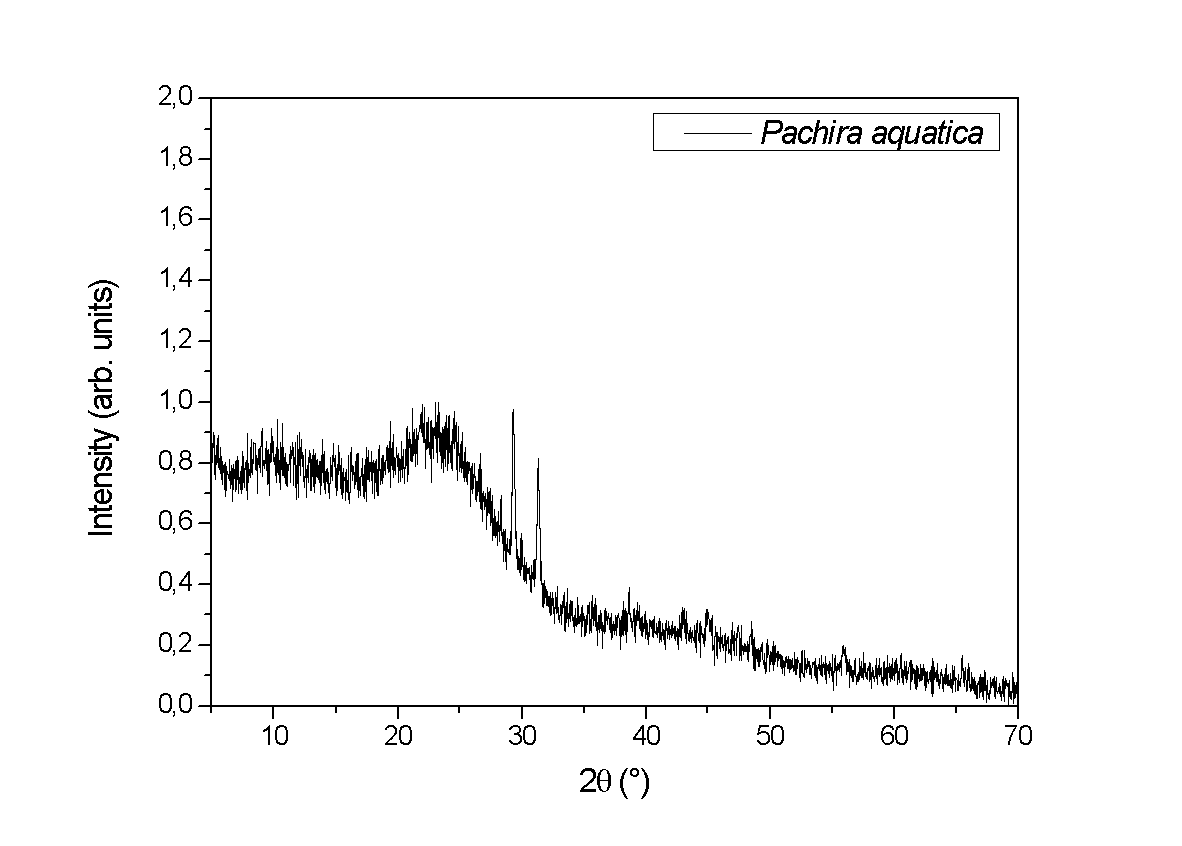


Fig. S.1. XRD pattern of the PAB

|  |  |
| --- | --- |

Fig S.2. Effects of factors on (a) **Ni**(II) (b) Cd(II) removal using PAB
